# *In vitro* application of *Eruca vesicaria* subsp. *sativa* leaf extracts and associated metabolites reduces the growth of *Oomycota* species involved in Kiwifruit Vine Decline Syndrome

**DOI:** 10.3389/fpls.2023.1292290

**Published:** 2023-12-18

**Authors:** Giovanni Mian, Kathryn Zuiderduin, Luke S. Barnes, Supasan Loketsatian, Luke Bell, Paolo Ermacora, Guido Cipriani

**Affiliations:** ^1^ Department of Agricultural, Food, Environmental and Animal Sciences, University of Udine, Udine, Italy; ^2^ Department of Crop Sciences, School of Agriculture, Policy & Development, University of Reading, Reading, Berkshire, United Kingdom

**Keywords:** *Eruca* spp., glucosinolates, isothiocyanates, liquid chromatography mass spectrometry, leaf extract, oomycetes, KVDS

## Abstract

This study aimed to determine whether leaf extracts from seven *Eruca vesicaria* subsp. *sativa* cultivars and their biochemically active compounds (glucosinolates and downstream-derived products) inhibit mycelia growth of three well-known pathogenic oomycetes, *Phytopythium chamaehyphon*, *Phytopythium vexans* and *Phytophthora citrophthora*; being the most significant in the development of Kiwifruit Vine Decline Syndrome (KVDS). Leaf extract quantity of 10, 20 and 30 mg were inoculated in Petri dish (90 mm Ø, each 22 mL of liquid medium – Potato Dextrose Agar), for *in vitro* bioassays. A pathogen plug was placed in the centre of each plate and the *Oomycota* colony perimeter was marked 5 days after inoculation. Radial colony growth was measured from 4 marks per plate 5, 10, and 15 days after inoculation, further elaborated with Image J software image analysis. Growth rates for all strains were inhibited by around 67% after 15 days. This was most pronounced when applying the highest concentration of leaf extract. By using Liquid Chromatography Mass Spectrometry (LC-MS) and Gas Chromatography Mass Spectrometry (GC-MS), fifteen glucosinolate compounds, of which glucosativin was found in the highest quantity, were identified. Concentrations of hydrolysis products produced by leaves (erucin and sativin) were also investigated, and were significantly associated with colony radial growth, especially towards *Pp. chamaehyphon* and *Pp*. *vexans*. Three downstream products of glucosinolates (two pure isothiocyanates, AITC and PEITC; and one indole I3C; all commonly present in Brassicaceae) were also tested, and a statistically significant inhibition of growth was observed at the highest concentration (0.6 µL).

## 1 Introduction

Kiwifruit is a recently domesticated plant belonging to the family *Actinidiaceae* and the genus *Actinidia*, which contains more than 54 species (Costa and Testolin, 2022). Consumption of kiwifruit has been increasing steadily, with the fruit recognised as being nutritious, owing to its high vitamin C content. It is a highly profitable crop with a long shelf life, making it suited to global trade (Mian et al., 2022a; Bassi et al., 2023). In Italy, the area planted with kiwifruit is currently 25,000 ha (repository http://dati.istat.it). Recently, a new and complex disease called Kiwifruit Vine Decline Syndrome (KVDS), has appeared in different kiwifruit growing regions, being reported initially in Italy and then in other countries (Donati et al., 2020; Türkkan et al., 2022). This has led to significant yield and economic losses in Italy, estimated to be around €300,000,000 in 2020 (Tacconi et al., 2019). The abiotic factor of waterlogging is thought to favour the development of this syndrome, although KVDS can occur in many soil types. Several soil‐borne pathogens have been implicated as aetiological agents (biotic factors). *Oomycota* species including *Phytophthora* spp. (*Phy*.), *Pythium* spp. (*P*.), and *Phytopythium* spp. (*Pp*.) have been frequently isolated from affected plants (Savian et al., 2020; Savian et al., 2022) and are thus candidates in causing KVDS. Pathogenicity on kiwifruit cuttings has been demonstrated for some isolates (e.g.*, Phy. citrophthora*, *Pp. vexans and Pp. chamaehyphon*) (Prencipe et al., 2020; Savian et al., 2021). The syndrome is complex, due to both biotic and abiotic factors. Furthermore, knowledge of the disease is still inadequate, and management strategies ineffective. The fungal kingdom comprises some of the most devastating plant pathogens (Möller and Stukenbrock, 2017), and is one of the major threats for crop cultivation. The *Oomycota* is a descendant of algal-like microorganisms, having a natural predisposition to parasitism (de Andrade Lourenço et al., 2020), and a life cycle which depends on water (Hargreaves and van West, 2019). They have specialized adaptations, which allow them to infect and kill many species of plants, some of which are important food and cash crops (Grenville-Briggs and Van West, 2005). The paradigmatic example is the highly destructive *Phy. infestans* or ‘potato blight’.

In 2022 it was reported the behaviour of *Actinidia* spp. germplasm grown in KVDS infected areas and identified resistance traits in *A. macrosperma* genotypes (Mian et al., 2022a; Mian et al., 2022b). However, integrating these genotypes into commercial production will necessitate either the adoption of rootstock, or incorporation into breeding practices. Additionally, the research evaluating the resistance efficacy *A. macrosperma*, as well as the impact of the rootstocks on fruit quality and yield, is still in its infancy. Therefore, the development of effective KVDS control using these methods is likely to be a long-term approach. A more immediate and promising approach to mitigate KVDS is through the use of biological agents, a practice already adopted to control several soil borne pathogens (Larkin and Griffin, 2007; Morales-Rodríguez et al., 2016; Cerritos-Garcia et al., 2021). This strategy is compatible with organic farming and does not increase environmental pollution (Poveda et al., 2020), also, there is the possibility to use compounds directly in early infected orchards and/or from the first year of cultivation, without having to uproot plants. There is an increasing pressure to develop low-input and more sustainable agricultural practices, including biological alternatives to synthetic chemicals for controlling pests and diseases (a major factor for heavy losses in agricultural production) (Bellostas et al., 2007; european-green-deal.it).

The family *Brassicaceae* contains approximately 372 genera and 4,060 species. It is considered an important plant family because it includes many economically important crops, such as kale, cabbage, and rocket. The group of crops collectively known as rocket (or arugula, rucola, roquette) are native to the areas surrounding the Mediterranean Sea (Martínez-Sánchez et al., 2006). Cultivated rocket crops comprise two genera, *Eruca* and *Diplotaxis*, which are increasingly important in the salad vegetable market. From the perspective of biological control, the *Brassicaceae* family has been studied extensively for sulfur-containing secondary metabolites, known as glucosinolates (GSLs) (Piślewska-Bednarek et al., 2018). Upon hydrolysis by endogenous enzymes (myrosinases) GSLs release a series of biologically active products with fungicidal, insecticidal, herbicidal and nematocidal properties (Bednarek et al., 2009). After hydrolysis, the unstable GSL aglycon (a thiohydroximate-*O*-sulfate) spontaneously rearranges to form various volatile and semi-volatile decomposition products. The most widely researched GSL decomposition products are the isothiocyanates (ITCs). These compounds are reactive towards nucleophiles (Hanschen et al., 2014), resulting in a wide array of pharmacological effects. As ITCs have potent antimicrobial activity, they have a significant effect on the soil microbiome and are also used in a variety of agricultural and food industry applications (Hu et al., 2015). ITCs are almost always claimed to be the most biologically active components of Brassicaceae, mainly due to their high reactivity toward electrophilic groups in biomolecules. This usage has been termed biofumigation. The term was coined in the early 1990s to describe the suppression of soil borne pests by volatile ITCs present in some *Brassica* crops (Kirkegaard and Sarwar, 1998; Khruengsai et al., 2021). Virtually all other members of the *Brassicaceae* contain GSLs as secondary metabolites that act as part of plant defence mechanisms. *Eruca vesicaria* subsp. *sativa* contains high levels of GSLs within the leaf tissue; the most prominent of which are glucosativin (4-mercaptobutyl-GSL; GSV), glucoerucin (4-(methylthio)butyl-GSL; GER) and glucoraphanin (4-(methylsulfinyl)butyl-GSL; GRA). GSV and GER breakdown products are thought to contribute to pungency and flavour in rocket (Bell et al., 2021). Numerous other GSLs have also been identified within rocket tissue, for example diglucothiobeinin (4-(b-D-glucopyranosyldisulfanyl)butyl-GSL; DGTB), 4-hydroxyglucobrassicin (4-hydroxy-3-indolymethyl-GSL; 4HB) and 4-methoxyglucobrassicin (4 methoxy-3-indolymethyl-GSL; 4MGB) (Bell et al., 2015; Yahya et al., 2019).

Previous studies have reported that GSL derived products (GL-DPs) were bioactive, and able to control nematodes (*in vitro*), fruit post-harvest pathogens, tumour cell proliferation, as well as entomopathogenic fungi; in fact, efficacy of biofumigation with *Brassica carinata* towards *Phy. cinnamoni* has been assessed (Morales-Rodríguez et al., 2016; Cerritos-Garcia et al., 2021). GSL derived volatile organic compounds (VOCs) have also been found to be effective for controlling soil-borne pathogens such as *P. ultimum* and *Fusarium oxysporum* and *graminearum*, in field plot studies (Gerik, 2005a; Gerik, 2005b; Ashiq et al., 2022). Poveda et al., (2020) reported the effective application of different *Brassica* green manure crops on the fungal pathogens *Sclerotinia minor*, *F. oxysporum* or *Rhizoctonia solani*, and the oomycete *P. dissotocum*. The effectiveness of indole-3-carbinol (I3C) towards several microbes (bacteria and fungi) was also assessed (Sung and Lee, 2007), and vapours of allyl-isothiocyanate (AITC) were evaluated in *in vitro* and *in vivo* trials against soil‐borne diseases in commercial tomato (Ren et al., 2018).

KVDS epidemiological traits are still under investigation, and at present there are no effective methods of control to stop the disease quickly spreading worldwide (Savian et al., 2022; Türkkan et al., 2022; Mian et al., 2022c), although some experience exists (Mian et al., 2023). Biofumigation for controlling soilborne pests is an indispensable practice for achieving consistent crop quality and yields during the commercial production of many vegetable and high value crops with mainly narrow rotation systems (Yu et al., 2019). As the use of soil fumigants is severely limited, the aim of our study was finding natural fungitoxic compounds as possible alternatives. Rocket was chosen as the species contains a high level of GSLs, related downstream hydrolysis products, and are fast-growing (Stajnko et al., 2022). Thus, to better understand and discover further candidate biocontrol agents, the objective of our study was to determine the *in vitro* inhibitory effects of leaf extracts of several *E. vesicaria* cultivars towards the oomycetes strain, identify the GSLs content and the GSL-downstream products, and finally the effectiveness of pure compounds (ITCs).

## 2 Materials and methods

### 2.1 Oomycetes species used in this study

The experiment used the following *Oomycota* species: *Pp. vexans* and *Pp. chamaehyphon*, belonging to the mycological collection of University of Udine and *Phy. citrophthora* isolate and kindly provided by Dr. B. Linaldeddu, University of Padua, Italy. These strains were isolated from KVDS infected kiwifruit plants and selected according to previous research which identified the microorganisms as having a putative key-role in KVDS occurrence (Tacconi et al., 2015; Prencipe et al., 2020; Savian et al., 2021; Savian et al., 2022). All pathogens were maintained at 10°C in a growing chamber prior to use, renewing the cultures every 15 days to ensure a consistent supply of fresh strains for every experiment.

### 2.2 Plant material

*Eruca* cultivars consisting of seven accessions (present on the market except where noted) were chosen: Astra (A), Sweet Flame (SF), Sweet Intensity (SI), Eruca16 (E), Victoria (V), Dentellata (D) and Uber (U). Astra was obtained from Tozer Seeds Ltd, Cobham, UK; Sweet Flame and Sweet Intensity were obtained from Elsoms Seeds Ltd., Spalding UK; Eruca16 from Warwick Genetic Resources Unit, University of Warwick, Warwick, UK; and Victoria, Dentellata and Uber from CN Seeds, Ely, UK. Three biological replicates of each accession/variety were germinated under controlled environmental conditions (in controlled environment cabinets) after being sown in a random sequence. The environmental conditions set up were: 16 hours day – 8 hours night, light intensity: ± 280 µmol m^2^ s^1^, day-time T°: 22°C, night-time T°: 14°C, relative humidity: 50-70%, and watering was performed by hand when necessary. Seedlings were grown for ten days in seedling trays and then transplanted to larger trays; four plants of each replicate were grown on. Plants were grown for another twenty days and then leaves from the four plants were harvested together. Sampling for each plant took approximately one minute from the cutting of the leaves at the petiole to being placed in zipper-top poly bag freezer bags on dry ice inside a polystyrene container (with lid). Thirty days was chosen as the optimum point of harvest as it reflects the typical number of days commercial growers grow their crop after sowing (Hall et al., 2012). Samples used for GSL and ITC extractions were placed in a -80°C freezer immediately after harvest and transport was completed (<30 min). Samples were freeze-dried in batches for three days (in a Vertis Bench-top Series Freeze Dryer-LaboGene™ CoolSafe freeze dryer) and milled to powder through using a Wiley Mini Mill (Swedesboro, New Jersey, USA).

### 2.3 Leaf extract preparation

Leaf extracts for use in oomycetes growth assays were prepared by using a slightly modified protocol from Cerritos-Garcia et al., 2021. Briefly, three biological replicates of each sample were prepared by adding 25 mL of 80% ethanol to 12.5 g of the lyophilised leaf powder (previously freeze dried and ground) and maintained at 20°C for one hour. The mixture was then vortexed for 15 seconds, before filtering through two layers of sterile gauze, and centrifuging at 4,000 RFC (Relative Centrifugal Force) for 15 min at 4°C to remove any tissue fragments. The supernatant was extracted and used to produce a 50 mL stock solution of 50% *w*/*v* (equivalent to 0.5 dry sample weight grams per mL) (**Figure S1A**). The stock solutions were then diluted to obtain the required treatment concentrations and immediately incorporated into the potato dextrose agar (PDA) (Difco™, Benton, Dickinson and Co., Sparks, MD, USA) plates to be used for *in vitro* bioassays, as soon as the temperature dropped to 30°C. Leaf extracts as dry weight, expressed as mg of leaves/Petri (i.e., mg of tested product/22 mL of PDA – Petri dish of 90 mm Ø), were tested at 10 mg, 20 mg, and 30 mg, recording values after 5, 10 and 15 days after inoculation.

### 2.4 Effect of leaf extracts on oomycetes growth

Following the method of Cerritos-Garcia et al. (2021), liquid medium (19.5 g of PDA powder/500 mL of sterile distilled water) was mixed in a 1 L bottle and autoclaved for 20 min at 121°C. After the liquid medium cooled to ~25°C, 50 mL of supernatant from one of the ethanol leaf extract concentrations were added and the medium was mixed again. Liquid medium (22 mL) containing the specific leaf extract concentration was poured into each of sterile plastic Petri dishes and allowed to solidify. For the control, 50 mL of 80% ethanol were added to the liquid medium (1 L) prior to pouring. Five biological replicates were used for each strain being tested with leaf extracts of each cultivar. Also, a first approach to all the pathogens together was set up. Due to the simultaneous nature of experiments, the biological replicates of controls were raised to seven. The PDA plates were inoculated by placing a plug (0.5 mm diameter) of the oomycetes on the centre of the plate at the intersection of the two perpendicular lines and sealed the Petri dish with Parafilm™ M (Bemis Co., Neenah, WI, USA) (**Figure S1B**). Petri dishes were placed in a growth chamber set at 23°C with a 14 h photo phase. The perimeter of the colony was marked on each perpendicular line on the underside of each plate and colony growth was assessed 5, 10, and 15 days after inoculation by measuring the colony radius from the crossing of the perpendicular lines to the perimeter of the colony in two perpendicular directions. The Radial Growth (RG) per time period was determined by the following formula: RG = (Rx *–* R0), where Rx = colony radius in mm at 5, 10, or 15 days after the initial colony radial measurement; R0 = initial colony radius in mm. After the measurements, we used the radius to set up the scale into ImageJ software (National Institutes of Health, USA) (Schneider et al., 2012), with which the total area (cm^2^) of *Oomycota* growth was determined. An example of the different pathogens and work is depicted in **Figures S2**–**S4**.

### 2.5 Effects of selected isothiocyanates on pathogens’ mycelium growth *in vitro* trial

Three different GSL hydrolysis products were used on the three pathogens: AITC (commonly found in mustards), phenethyl isothiocyanate (PEITC; commonly found in watercress and other *Brassicaceae* species), and indole 3-carbinol (I3C; commonly found in *Brassicaceae* species – a degradation product of the unstable ITC which is formed from glucobrassicin and other indolic GSLs). The standards (>98%) were obtained from Merck-Sigma (Gillingham, UK). Following Ugolini et al., 2014, in each case, different aliquots of pure compounds (0.30 and 0.60 µL × 90 mm diameter Petri dishes) were placed, using a micro syringe, on a 90 mm diameter paper filter (Whatman No. 1), positioned inside the cover. The dishes were quickly closed, sealed by Parafilm, and incubated at 23°C. The dishes were opened after 3 days to remove the paper filter, as after this point the volatile compounds have generally saturated their effect. Furthermore, the fungicidal activity of AITC was evaluated, after 5 days (first evaluation); then 10 and 15 days. Petri dishes inoculated with the pathogen but treated with distilled water in place of ITCs were used as a control. Five samples of one dish were used for each treatment (different ITCs concentrations) and the experiment was repeated twice. As concentrations, 0.3 µL of AITC is 1.37 x 10^-7^ M while 0.6 µL stays for 2.75 x 10^-7^ M. Considering PEITC, 0.3 µL is equivalent to 1.01 x 10^-7^ M and 0.6 µL to 2.02 x 10^-7^ M. Lastly, regarding I3C, the concentrations applied were 9.27 x 10^-8^ M and 1.85 x 10^-7^ M, respectively for 0.3 and 0.6 µL. Mycelium growth inhibition was evaluated as aforementioned.

### 2.6 Glucosinolate extraction

GSL extractions were prepared according to Jasper et al. (2020), with slight modifications. Forty mg of dried leaf powder were obtained after the freeze drying of fresh leaves, Ground, and put into Eppendorf tubes and placed in a heat block at 80°C for ten minutes before adding 1 mL of preheated 70% (v/v) methanol. Samples were vortexed vigorously and placed in a water bath (70°C for 10 min). Samples were allowed to cool then centrifuged at 12,500 x *g*, at room temperature for five minutes. The supernatant was collected using a 1 mL syringe and filtered using a PVDF 0.22 µm syringe filter (VWR, Lutterworth, UK). Three biological replicate samples were prepared for each cultivar. Extracts were stored at -80°C before dilution (5x) with distilled water and analysis conducted by LC–MS.

### 2.7 LC–MS analysis

All solvents and chemicals used were of LC–MS grade and obtained from Sigma–Aldrich (Poole, UK) unless otherwise stated. Authentic standards were purchased from PhytoPlan (Heidelberg, Germany): glucoiberin (GIB; 99.61%), progoitrin (PRO; 99.07%), glucoraphanin (GRA; 99.86%), sinigrin (SIN; 99%), glucoalyssin (GAL; 98.8%), gluconapin (GNP; 98.66%), 4-hydroxyglucobrassicin (4HGB; 96.19%), glucotropaeolin (GTP; 99.61%), glucoerucin (GER; 99.68%), glucobrassicin (GBC; 99.38%), gluconasturtiin (GNT; 98.38%), 4-methoxyglucobrassicin (4MGB; 94.78%), neoglucobrassicin (NGB; 99.13%). All compound purities were determined by High Performance Liquid Chromatography Diode Array Detector (HPLC-DAD). Diglucothiobeinin (DGTB), glucosativin (GSV), and dimeric 4-mercaptobutyl GSL (DMB) were semi-quantified using SIN as no authentic standards were available. A dilution series of concentrations was prepared as external calibration curves with HPLC-grade water.

UPLC-MS was performed on a Shimadzu Nexera X2 series UHPLC, coupled with an 8050 triple quadrupole mass spectrometer system (Shimadzu UK Ltd., Milton Keynes, UK), following the method of Bell et al. (2021). Separation of compounds was achieved using a Acquity UPLC BEH C18 1.7 μm column (Waters Corporation, Milford, USA) Mobile phases consisted of 0.1% formic acid in LC-MS grade H_2_O (A), and 0.1% formic acid in LC-MS grade acetonitrile (B). GSLs were separated during a five minute run with the following gradient timetable: (i) 0–50 s (A-B, 98:2, *v/v*), (ii) 50 s–3 min (A-B, 70:30, *v/v*), (iii) 3–3 min 10 s (A-B, 5:95, *v/v*), (iv) 3 min 10 s–4 min (A-B, 5:95, *v/v*), (v) 4–4 min 10 s (A-B, 98:2, *v/v*), (vi) 4 min 10 s–5 min (A-B, 98:2, *v/v*). The flow rate was 0.4 mL per min and the column oven temperature was 35°C.

Two MS methods were used for the identification and quantification of GSLs. First, a Product Ion Scan (PIS) method was established to identify GSLs based on known primary ion masses ([M-H]-) characteristic fragment ions (*m/z* 357, 258, and 97; Bell et al., 2021). Then, MS/MS spectra were compared to authentic standards. MS/MS settings for the PIS method were as follows: samples were analysed in the negative ion mode with a mass scan range of 70–820 amu. A collision energy of 25 eV and a scan speed of 30,000 u per s^−1^. For the quantification of GSLs, a Multiple Reaction Monitoring (MRM) method was used (Bell et al., 2021).

### 2.8 Isothiocyanate (sativin and erucin) extraction & analysis by GC-MS

The major hydrolysis products produced by *Eruca* leaves, sativin and erucin, were investigated and extracted according to the protocol of Bell et al. (2020). GC–MS was performed on an Agilent 7693/5975 GC–MS with autosampler (Agilent, Manchester, UK). 1 μL of each sample was injected into VF-5ht 15 m column (0.1 μm film thickness, 0.25 mm I.D.; Agilent). Injection temperature was 250°C in split mode (1:20); oven temperature was programmed from to 40°C at a rate of 5°C/min until 320°C. Carrier gas was helium, with a flow rate of 1.1 mL/min and a pressure of 1.4 psi. Mass spectra were obtained by electron ionization at 70 eV, and mass scan from 35 to 500 amu. Compounds were identified using literature ion data and quantified based on integrated peak areas of an external standard calibration curve of AITC (Sigma). Five concentrations of AITC were prepared from a stock of in dichloromethane (DCM), ranging from 0 to 10 μM (r^2^ = 0.999; y = 1E+06). Data analysis was performed using ChemStation for GC–MS (Agilent). Data are expressed in nmol g^-1^ dry weight.

### 2.9 Statistical analyses

Shapiro–Wilk normality test was conducted for all analyses. All tests were concluded to fit with a normal distribution and allow for statistical comparison using a parametric test. One‐way Analysis Of VAriance (ANOVA) was performed by using “R” software (version 4.0.3 2020‐10‐10). Statistical analysis to determine significant differences between treatment means was carried out using a protected *post-hoc* Tukey’s honest significant difference (HSD) test (*p*-values < 0.05). The R library ‘multicomp letters’ was applied to assigned unique letters to denote significant difference; for boxplots, the library ‘ggplot2’ was used. Lastly, for the Pearson correlation index between variables, the R library ‘corrplot’ was used (Colautti et al., 2023).

## 3 Results

### 3.1 Effect of leaf extracts and ITCs on oomycetes growth

Cv. Astra leaf extracts demonstrated the capability to reduce *Pp. chamaehyphon* growth in all three treatment concentrations, with the most pronounced effects being observed at 5 Days After Inoculation (DAI), especially at 30 mg. At 15 days, the 30 mg treatment reduced growth by approximately 75% relative to the control, with similar trends observed for *Pp. vexans* and *Phy. citrophthora* (76% and 87%, respectively; **Table 1**).

**Table 1:** Means of three Oomycota area growth (cm^2^) grown on PDA with seven rocket leaf extracts (5 cultivars.) at four concentrations (0-check, 10, 20 and 30 mg), recorded after 5-, 10- and 15-days following administration of the extract.

Pathogens													
		*Pp. chamaehyphon*				*Pp. vexans*				*Phy. citrophthora*			
		Treatments (mg)											
Cultivars	Days after inoculation	Check	10	20	30	Check	10	20	30	Check	10	20	30
**Astra**	5	14.80 a	5.55 b (62.5)	4.82 b (67.43)	2.48 c (83.24)	15.33 a	7.25 b (52.70)	8.54 b (44.29)	6.34 b (58.64)	4.10 a	2.32 b (43.41)	0.38 c (90.73)	0.28 c (93.17)
	10	26.22 a	15.61 b (40.46)	12.56 b (52.10)	9.82 c (62.55)	40.24 a	11.09 b (72.44)	11.00 b (72.66)	10.05 b (75.02)	7.06 a	3.46 b (50.99)	2.00 b (71.67)	0.91 c (87.11)
	15	62.45 a	19.62 b (68.58)	17.63 b (71.77)	15.00 b (75.98)	61.90 a	19.15 b (69.06)	18.84 b (69.55)	14.32 c (76.87)	24.10 a	7.64 b (68.30)	4.29 b (82.20)	2.95 c (87.76)
**Sweet** **intensity**	5	14.80 a	13.97 a (5.61)	12.18 a (17.70)	12.56 a (15.14)	15.33 a	9.94 b (35.16)	9.50 b (38.03)	8.65b (43.57)	4.10 a	5.30 b (0)	2.43 c (40.73)	0.15 d (93.34)
	10	26.22 a	23.57 a (10.11)	19.46 b (25.78)	17.19 b (34.44)	40.24 a	31.15 b (22.59)	25.1 b (37.62)	26.77 b (33.47)	7.06 a	7.35 a (0)	6.78 a (3.97)	0.22 b (96.88)
	15	62.45 a	30.76 b (50.74)	24.26 b (61.15)	26.77 b (57.13)	61.90 a	26.04 b (57.93)	22.89 b (63.02)	24.61 b (60.24)	24.10 a	10.63 b (55.89)	9.39 b (91.04)	3.06 c (87.30)
**Dentellata**	5	14.80 a	17.04 a (0)	14.78 a (0.14)	15.33 a (0)	15.33 a	11.45 b (25.31)	9.83 b (35.88)	10.74 b (24.94)	4.10 a	0.19 b (95.37)	0.78 b (80.98)	0.08 c (98.05)
	10	26.22 a	28.63 a (0)	23.74 b (9.46)	20.90 b (20.29)	40.24 a	14.92 b (62.92)	15.33 b (65.98)	12.68 b (66.51)	7.06 a	8.86 b 0	6.55 b (39.79)	4.29 c (39.24)
	15	62.45 a	37.59 b (39.81)	32.75 b (47.56)	29.57 b (52.65)	61.90 a	21.38 b (65.46)	21.06 b (65.98)	20.73 b (66.51)	24.10 a	15.47 b (35.81)	14.51 b (39.79)	9.21 c (61.78)
**Victoria**	5	14.80 a	12.18 a (17.70)	11.81 a (20.20)	10.98 a (25.81)	15.33 a	13.19 b (13.96)	11.33 b (26.09)	12.43 b (18.92)	4.10 a	0.32 b (92.20)	0.78 b (80.98)	0.55 b (86.59)
	10	26.22 a	20.57 a (21.55)	16.64 b (36.54)	14.92 b (43.10)	40.24 a	17.19 b (57.28)	17.63 b (59.19)	14.64 b (63.62)	7.06 a	7.54 a (0)	6.87 a (2.69)	0.98 b (86.12)
	15	62.45 a	26.77 b (57.13)	21.22 b (66.02)	23.40 b (62.53)	61.90 a	24.61 b (60.24)	24.26 b (60.81)	23.90 b (61.39)	24.10 a	19.62 b (18.59)	14.51 b (39.79)	7.06 c (70.71)
**Sweet flame**	5	14.80 a	8.75 b (40.87)	7.35 b (50.34)	6.24 b (57.84)	15.33 a	10.74 b (29.94)	7.44 b (51.47)	5.97 b (61.06)	4.10 a	4.86 a (0)	2.37 b (42.20)	0.78 c (80.98)
	10	26.22 a	12.81 b (51.14)	10.86 b (58.58)	7.96 b (69.64)	40.24 a	15.19 b (62.25)	11.45 b (71.55)	10.06 b (75)	7.06 a	8.13 a (0)	5.97 b (15.44)	4.59 b (34.99)
	15	62.45 a	20.41 b (67.32)	15.60 b (75)	13.06 b (70.08)	61.90 a	21.72 b (64.91)	19.20 b (68.98)	15.67 b (74.68)	24.10 a	15.19 b (36.97)	11.41 b (52.66)	11.34 b (52.95)

Within columns, values assigned with different letters are significantly different (Tukey’s HSD test, *p <* 0.05). Between brackets: % of inhibition ((check area-treatment area/check area))*100.

In the case of cv. Sweet Intensity and *Pp. chamaehyphon*, no statistical effects were observed at 5 DAI for each applied concentration. However, at 10 DAI, concentrations of 20 and 30 mg exhibited growth reduction. At 15 DAI, all concentrations demonstrated a statistically significant reduction in growth (*p* < 0.05). At 15 days and with a 30 mg application, the growth reduction was approximately 57% relative to the control. Similar patterns were observed for *Pp. vexans* and *Phy. citrophthora*, with varying concentrations and DAI affecting growth reduction, reaching approximately 60% and 87% reduction, respectively, at 15 days and with a 30 mg application (**Table 1**).

Considering cv. Dentellata and *Pp. chamaehyphon*, concentrations of 10, 20, and 30 mg did not influence growth at 5 DAI, nor did 10 mg at 10 DAI. However, a statistically significant (*p* < 0.05) reduction was observed for the remaining combinations, notably at 15 DAI with a 30 mg application, resulting in a reduction of approximately 52% relative to the control. For *Pp. vexans*, each concentration at every DAI statistically reduced growth, with a 66% reduction at 15 days and with a 30 mg application. Lastly, for *Phy. citrophthora*, each concentration exhibited an effect, particularly noticeable at 30 mg at each DAI. At 15 days and with a 30 mg application, the reduction in growth was approximately 62% relative to the control (**Table 1**).

Cv. Victoria leaf extracts of all three concentrations did not have an effect on *Pp. chamaehyphon* growth at 5 DAI. At 10 DAI, only 10 mg did not result in any reduction. However, for the remaining combinations, all concentrations at each DAI caused a reduction in mean growth compared with the control (*p* < 0.05), although differences were not statistically significant between treatments (*p* < 0.05). At 15 days and with a 30 mg application, the growth reduction was approximately 62% relative to the control. Similar patterns were observed for *Pp. vexans* and *Phy. citrophthora*, with varying concentrations and DAI affecting growth reduction, reaching approximately 61% and 70% reduction, respectively, at 15 days and with a 30 mg application (**Table 1**).

Cv. Sweet Flame extracts reduced the growth of *Pp. chamaehyphon* in all treatments compared with the control, with no significant differences being observed between treatments (*p* < 0.05). At 15 days with a 30 mg application, there was approximately a 70% reduction relative to the control. This trend was mirrored for *Pp. vexans*, where at 15 days and with a 30 mg application, the reduction in growth was approximately 75% relative to the control. For *Phy. citrophthora*, 10 mg at 5 and 10 DAI did not show any effect, while the remaining concentrations demonstrated a statistical reduction, particularly noticeable at 30 mg after 5 DAI. At 15 days and with a 30 mg application, the growth reduction was approximately 53% relative to the control (**Table 1**).

Taking into consideration cv. Eruca16 and *Pp. chamaehyphon*, each concentration at each DAI demonstrated a reduction in oomycete growth, particularly pronounced at 5 DAI with 30 mg. At 15 days and with a 30 mg application, the reduction in growth was approximately 75% relative to the control. Similar patterns were observed for *Pp. vexans*, with each concentration at each DAI resulting in a reduction in oomycete growth. At 15 days and with a 30 mg application, the reduction in growth was approximately 74% relative to the control. Lastly, for *Phy. citrophthora*, 10 mg at 5 DAI did not show any effect, but for the remaining combinations, each one statistically reduced growth, particularly noticeable at 30 mg at each DAI. At 15 days and with a 30 mg application, the reduction in growth was approximately 78% relative to the control (**Table 2**).

**Table 2:** Means of three *Oomycota* area growth (cm^2^) grown on PDA with seven rocket leaf extracts (2 cultivars.) at four concentrations (0-check, 10, 20 and 30 mg), recorded after 5-, 10- and 15-days following administration of the extract.

Pathogens														
		*Pp. chamaehyphon*				*Pp. vexans*					*Phy. citrophthora*			
		Treatments (mg)												
Cultivars	Days After inoculation	Check	10	20	30	Check	10	20	30	Check		10	20	30
**Eruca16**	5	14.80 a	6.06 b (59.05)	5.63 b (61.96)	2.01 c (86.42)	15.33 a	9.50 b (38.03)	8.13 b (46.97)	8.96 b (41.55)	4.10 a		4.15 a (0)	2.32 b (43.41)	0.22 c (96.63)
	10	26.22 a	11.45 b (56.33)	13.58 b (48.21)	11.33 b (56.79)	40.24 a	12.43 b (69.11)	12.68 b (68.49)	10.51 b (73.88)	7.06 a		4.16 b (41.08)	4.64 b (34.28)	0.88 c (87.54)
	15	62.45 a	15.89 b (74.56)	16.18 b (74.09)	15.33 b (75.45)	61.90 a	17.78 b (71.28)	17.48 b (71.76)	15.78 b (74.51)	24.10 a		15.61 b (35.23)	15.33 b (36.39)	5.14 c (78.67)
**Uber**	5	14.80 a	13.06 a (11.76)	12.68 a (14.32)	11.69 a (21.01)	15.33 a	11.09 b (27.66)	9.50 b (38.03)	10.51 b (31.44)	4.10 a		0.72 b (82.44)	0.94 b (77.07)	0 c (100)
	10	26.22 a	22.05 a (15.90)	18.23 b (30.46)	16.08 b (38.67)	40.24 a	14.64 b (63.62)	14.92 b (62.92)	12.31 b (69.41)	7.06 a		5.97 b (15.44)	6.42b (9.07)	4.15 b (41.22)
	15	62.45 a	28.82 b (53.85)	22.72 b (63.62)	25.14 b (59.74)	61.90 a	20.90 b (66.24)	20.57 b (66.77)	20.25 b (67.29)	24.10 a		22.21 a (7.84)	13.32 b (44.73)	12.56 b (44.88)

Within columns, values assigned with different letters are significantly different (Tukey’s HSD test, *p* < 0.05). Between brackets: % of inhibition (check area-treatment area/check area)*100.

Lastly, Cv. Uber, and considering *Pp. chamaehyphon*, the concentration at 5 days did not show an effect, nor did 10 mg at 10 DAI. For the remaining combinations, there was a statistically significant reduction in growth but without a difference among them. At 15 days and with a 30 mg application, the reduction in growth was approximately 60% relative to the control. For *Pp. vexans*, each concentration at each DAI resulted in a reduction in oomycete growth, without statistical significance among them. At 15 days and with a 30 mg application, the reduction in growth was approximately 67% relative to the control. Lastly, for *Phy. citrophthora*, only 10 mg at 15 DAI did not show a statistically significant reduction. The remaining combinations showed a statistically significant oomycete reduction, particularly noticeable at 30 mg at 5 DAI. At 15 days and with a 30 mg application, the reduction in growth was approximately 44% relative to the control (**Table 2**).

In the context of AITC and *Pp. chamaehyphon*, each concentration applied at every recording stage significantly reduced oomycete growth, with the most pronounced effect observed at 0.6 µL on days 10 and 15 after inoculation (DAI), in comparison to the control. At 15 DAI, the percentage of inhibition was approximately 96% with 0.6 µL (**Table 3**).

**Table 3:** Means of three *Oomycota* area growth (cm^2^) grown on PDA with three ITCs (AITC, I3C and PEITC) at three concentrations (0-check, 0.3 and 0.6 µL), recorded after 5, 10 and 15 days after the administration of leaf extract.

		Pathogens								
		*Pp. chamaehyphon*			*Pp. vexans*			*Phy. Citrophthora*		
		Treatments (µL)								
GSL	Days after inoculation	check	0.3 µL	0.6 µL	check	0.3 µL	0.6 µL	check	0.3 µL	0.6 µL
AITC	5	8.54 a	0 b (100)	0 b (100)	3.79 a	0.00 b (100)	0.00 b (100)	3.33 a	0 b (100)	0 b (100)
	10	28.26 a	3.79 b (86.56)	0.78 c (97.22)	8.96 a	0.08 b (99.10)	0 b (100)	10.81 a	0.05 b (99.50)	0 b (100)
	15	55.38 a	10.28 b (81.43)	1.76 c (96.81)	31.35 a	9.07 b (71.06)	4.52 b (85.58)	20.57 a	1.67 b (91.86)	0.03 b (99.85)
I3C	5	8.54 b	17.78 a (0)	18.38 a (0)	3.79 a	0.75 b (80.96)	0.72 b (81.15)	3.33 a	2.89 a (7.84)	2.71 a (13.51)
	10	28.26 a	25.68 a (1.66)	28.16 a (0)	8.96 a	5.15 b (42.64)	1.53 c (82.84)	10.81 a	9.39 a (12.55)	6.06 a (43.55)
	15	55.38 a	58.32 a (0)	45.48 b (17.71)	31.35 a	25.14 b (19.80)	14.51 b (53.71)	20.57 a	19.00 a (7.66)	15.75 b (18.58)
PEITC	5	8.54 a	1.81 b (78.78)	0 b (100)	3.79 a	0 b (100)	0 b (100)	3.33 a	0 b (100)	0 b (100)
	10	28.26 a	15.47 b (45.24)	0 c (100)	8.96 a	0 b (100)	0 b (100)	10.81 a	0.032 b (99.71)	0 b (100)
	15	55.38 a	20.61 b (62.56)	3.20 c (94.22)	31.35 a	9.61 b (69.35)	1.36 c (95.66)	20.57 a	0.34 b (98.34)	0.28b (98.63)

Within columns, values assigned with different letters are significantly different at (Tukey’s HSD test, *p* < 0.05). Between brackets: % of inhibition ((check area-treatment area/check area))*100.

Concerning *Pp. vexans*, both concentrations demonstrated a reduction in growth compared to the control, though without statistical significance between them. At 15 DAI, the percentage of inhibition with 0.6 µL was approximately 85%. Similar trends were observed for *Phy. citrophthora*, where at 15 DAI, the percentage of inhibition with 0.6 µL was approximately 99% (**Table 3**).

In the case of I3C and *Pp. chamaehyphon*, only the application of 0.6 µL at 15 DAI exhibited a statistically significant reduction in oomycetes compared to the control, with a percentage of inhibition of approximately 17% (*p* < 0.05). For *Pp. vexans*, each concentration resulted in a reduction in growth, particularly notable at 10 DAI with 0.6 µL. At 15 DAI, the percentage of inhibition with 0.6 µL was approximately 53%. As for *Phy. Citrophthora*, only 0.6 µL at 15 DAI statistically inhibited growth, with a percentage of inhibition of approximately 18% (**Table 3**).

Shifting focus to PEITC and *Pp. chamaehyphon*, each concentration applied significantly reduced oomycetes growth, with the most pronounced effect observed at 0.6 µL at every DAI. At 15 DAI, the percentage of inhibition with 0.6 µL was approximately 94% (*p* < 0.05). For *Pp. vexans*, there was a statistically significant reduction in growth at each concentration applied, with a percentage of inhibition of approximately 95% at 15 DAI with 0.6 µL. Finally, considering *Phy. citrophthora*, both concentrations reduced growth compared to the control, with no significant difference among the concentrations. At 15 DAI, the percentage of inhibition with 0.6 µL was approximately 98% (**Table 3**).

### 3.2 Glucosinolate identification and quantification

In total 15 different GSLs compounds were detected and identified in *Eruca* leaves. Major compounds present were GSV, GER and DGTB. All other compounds were present in similar quantities. The highest total concentration was found in SF, followed by V, D, and U, which contained similar abundances and significantly lower than SF. A, E, and SI were the cultivars with the lowest concentrations, not being statistically different from each other (**Table 4**). Given these results, we calculated the GSL concentration present in each cv. applied at the different inoculation levels (10, 20 and 30 mg), confirming the results of the GSL sum. Results are listed in **Table 5**.

**Table 4:** Glucosinolates identified and quantified, expressed as µmol g^-1^ dry weight.

Cultivar							
GSL (µmol g^-1^ dry weight^)^	A	E	SI	SF	D	U	V
PRO	0.004	0.004	0.002	0.002	0.002	0.004	0.002
GRA	0.769	1.415	1.547	1.739	1.184	1.273	1.693
GNP	0.002	0.003	0.002	0.003	0.007	0.009	0.012
4HB	0.271	0.124	0.077	0.030	0.185	0.250	0.318
GTP	0.476	0.285	0.415	0.717	0.588	0.353	0.513
GBC	0.013	0.016	0.011	0.012	0.012	0.015	0.010
GNT	0.150	0.161	0.137	0.097	0.162	0.174	0.148
GIB	0.002	0.005	0.004	0.007	0.006	0.007	0.009
GSV	38.224	25.263	37.835	53.889	43.216	45.727	48.232
4MGB	0.844	1.359	1.089	1.038	0.075	0.070	0.078
NGB	0.845	1.339	1.078	1.034	0.470	0.484	0.521
DGTB	1.246	2.185	0.615	0.601	8.820	9.407	10.960
DMB	0.631	0.327	0.664	1.224	0.737	0.644	0.599
GAL	0.021	0.040	0.027	0.034	0.201	0.214	0.234
GER	19.535	19.516	20.500	22.295	16.339	16.323	17.146
**Total** **∑**	63.033 b	52.042 bc	64.003 b	82.722 a	72.004 ab	74.954 ab	80.475 a

Within the sum row, values assigned by different letters are significantly different at (Tukey’s HSD test, *p* < 0.05). GSL, glucosinolate; PRO, progoitrin; GRA, glucoraphanin; GNP, gluconapin; 4HB, 4-hydrocyglucobrassicin; GTP, glucotropaeolin; GBC, glucobrassicin; GNT, gluconasturtiin; GIB, glucoiberin; GSV, glucosativin; 4MGB, 4-methoxyglucobrassicin; NGB, neoglucobrassicin; DGTB; diglucothiobeinin; DMB, dimeric 4-mercaptobutyl GSL; GAL, glucoalyssin; GER, glucoerucin.

**Table 5:** Total GSL concentrations (µmol) present in each amount of inoculum applied to pathogens (10, 20 and 30 mg) per cultivar.

	10 mg	20 mg	30 mg
**Astra**	0.63	1.26	1.89
**Sweet Intensity**	0.64	1.28	1.92
**Dentellata**	0.72	1.44	2.16
**Victoria**	0.80	1.61	2.41
**Sweet Flame**	0.83	1.65	2.48
**Eruca16**	0.52	1.04	1.56
**Uber**	0.75	1.50	2.25

### 3.3 Erucin and sativin detection and quantification

**Figure 1:**
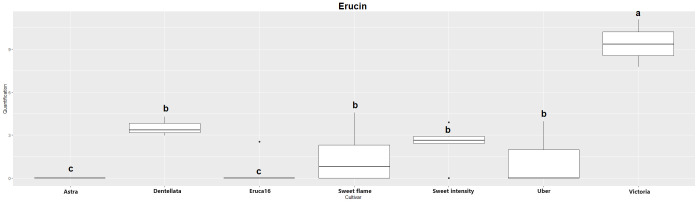
Boxplot of erucin quantification (uM) in the samples. Cv. Astra, Dentellata, Eruca16, Sweet Flame, Sweet Intensity, Uber and Victoria. Plots assigned by different letters are statistically significant at *p* < 0.05, applying the Tukey HSD test.

**Figure 2:**
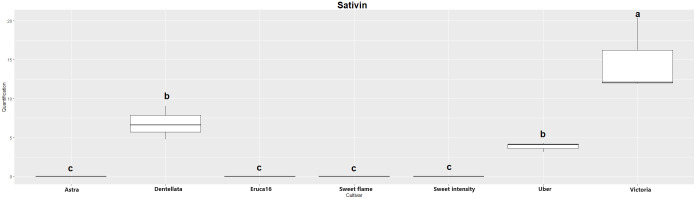
Boxplot of sativin quantification (uM) in the samples. Cv. Astra, Dentellata, Eruca16, Sweet Flame, Sweet Intensity, Uber and Victoria. Plots assigned by different letters are statistically significant at *p* < 0.05, applying the Tukey HSD test.

The two major hydrolysis products of *Eruca* spp. leaves were investigated, however, were not detectable in all samples. Erucin was not detected in cultivars E and A, for example. Contrastingly, erucin was identified in SF, SI, D, U, and V cultivars. Notably, V produced the highest concentrations of this compound, significantly surpassing all other instances. Conversely, no statistically significant distinctions emerged among SF, SI, D, and U cultivars (**Figure 1**). Sativin was detected and semi-quantified in V, U, and D. Similarly, V produced the most significant abundance of the compound, followed by U and D (with no statistically meaningful differentiation between the latter two; **Figure 2**). Adding a layer of complexity to the analysis, when considering the cultivars where both erucin and sativin compounds were identified and semi-quantified (D, U, and V), a correlation between their abundances is apparent. To substantiate this observation, the correlation coefficient (r^2^) was computed, yielding a value of 0.714.

### 3.4 Correlation existing between pathogens’ radial growth (at 15 days) and GSL content in the tested cultivars

**Figure 3:**
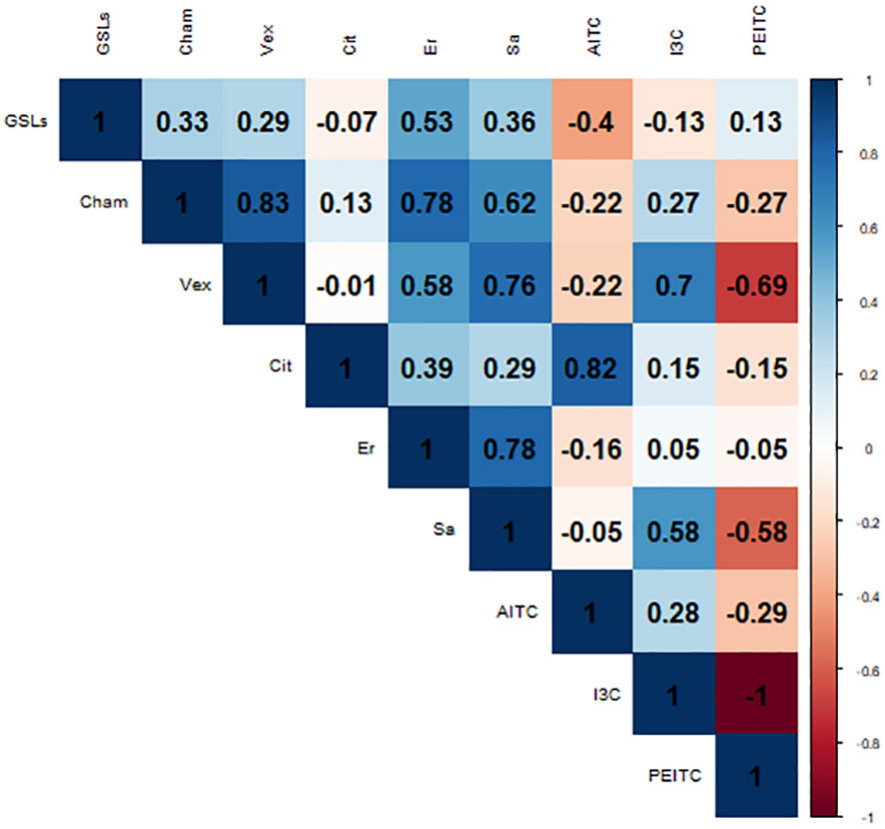
Pearson correlation index between the oomycetes area growth after 15 days -Cham: *Pp. chamaehyphon*, Vex: *Pp. vexans*, Cit: *Phy. citrophthora*-, the GSLs; erucin (Er) and sativin (Sa) content of each rocket cultivar tested, and the pure compounds adopted (ITC, I3C and PEITC).

The Pearson correlation coefficient (r^2^) has been employed to quantify the relationship between the radial growth of oomycetes after a 15-day period and the cumulative content of GSLs, erucin, and sativin within the cultivar under investigation and the pure compounds applied at the highest concentration (AITC, I3C and PEITC) (**Figure 3**). This index operates within a spectrum ranging from -1 to +1, encapsulating the extent of correlation. In the context of this study, we observed a correlation that, although modest, bears a positive trend. Regarding the total GSL content and its effect on growth inhibition, *Pp. chamaehyphon* displayed a correlation coefficient of 0.33. A positive correlation of 0.29 was deduced for *Pp. vexans*, while no discernible correlation surfaced in the case of *Phy. citrophthora* in relation to GSL concentrations (-0.07).

Focusing on erucin and sativin, *Pp. chamaehyphon* produced the highest positive correlation, yielding coefficients of 0.78 for erucin and 0.62 for sativin. Similarly, a positive correlation was established for *Pp. vexans*, exhibiting coefficients of 0.58 for erucin and 0.76 for sativin. Correspondingly, *Phy. citrophthora* exhibited a positive correlation as well, albeit weaker in comparison to *Pp. chamaehyphon* and *Pp. vexans*, registering coefficients of 0.39 for erucin and 0.29 for sativin.

When examining the pure compounds, a notable and robust correlation emerged exclusively between *Phy. citrophthora* and AITC, exhibiting a coefficient of 0.82. Similarly, *Pp. vexans* displayed a significant correlation with I3C, marked at 0.7. Conversely, in the remaining cases, correlations were observed to be quite weak, displaying both positive and negative trends. This contrasts sharply with the findings related to downstream products, erucin and sativin, where the correlation with radial growth inhibition is notably stronger, on average.

## 4 Discussion

In the current research seven accessions of rocket (*Eruca* spp.) extracts were tested at three different concentration levels against three oomycetes pathogens, which have a primary role in KVDS. In addition, three GSL pure compounds were also tested (i.e., ITCs and an indole), at two different concentrations. The cultivars being tested showed only limited differences when compared with each other, in terms of the effect they had on the growth of the *Oomycota* tested.

However, more in detail, regarding cv. Astra and *Pp. chamaehyphon*, all concentrations were effective, with the most significant impact at 5 and 10 DAI. A 30 mg application at 15 days resulted in a substantial 75% reduction in growth (on average). A consistent trend found with similar outcomes observed for the other cultivars (SI, D, V, SF, E, and U) and pathogens considered. With 30 mg applications at 15 days there were substantial reductions in growth for each pathogen, ranging from 60% to 78%. In conclusion, considering the highest application at 15 DAI for each cultivar and pathogen, on average, the reduction in oomycete growth was approximately 67% relative to the control, hence, the *Eruca* leaf extracts worked effectively. Similar results are already reported in literature for oomycetes and fungi (Kirkegaard and Sarwar, 1998; Charron and Sams, 1999; Sharma et al., 2018; Cerritos-Garcia et al., 2021). This is an important outcome: the evidence suggests that the effectiveness on the inhibition of growth did not slow and remained at a good level. Hence, the outcomes are of great interest for future application *in vivo*, where a community of pathogens (i.e., pathobiome) are present, and which lead to KVDS. In future work we aim to establish if the same effects of *Eruca* leaf extracts can be observed. Thus, this study suggests promising results for managing oomycete growth, emphasizing the importance of specific concentrations and application timings for different cultivar-pathogen combinations.

Comparing the effects of pure compounds, AITC significantly reduced *Pp. chamaehyphon* growth by 96% with 0.6 µL (10 and 15 DAI) and may constitute an effective control. Similarly, *Pp. vexans* exhibited an 85% growth inhibition at 15 DAI with 0.6 µL. A robust growth inhibition of approximately 99% at 15 DAI with 0.6 µL was observed for *Phy. citrophthora*. For I3C and *Pp. chamaehyphon*, a significant reduction of 17% occurred with 0.6 µL at 15 DAI. Likewise, for *Pp. vexans*, there was a notable 53% inhibition at 15 DAI with 0.6 µL. In the case of *Phy. citrophthora*, a statistically significant growth inhibition of approximately 18% was observed with 0.6 µL at 15 DAI. Indeed, considering PEITC and *Pp. chamaehyphon*, there was a significant reduction with each concentration, most notably at 0.6 µL (94% inhibition at 15 DAI). This trend was mirrored in *Pp. vexans* (95% inhibition at 15 DAI with 0.6 µL) and *Phy. citrophthora* (both concentrations exhibited growth reduction, with approximately 98% inhibition at 15 DAI with 0.6 µL). In conclusion, all isolated ITCs were effective, especially in the case of AITC and PEITC *versus* all oomycetes considered, whilst the activity of I3C was less effective. Moreover, we can state how the activity of these compounds is both of fungistatic and fungitoxic, as until they are removed, the pathogens do not grow. For this, our visual inspections did not reveal a growth until the removing, and after that, the pathogens started to grow again. Measurable effects at only 5 days after the application were possible. This work has demonstrated the effectiveness of other common ITC hydrolysis products and suggests that it is their action responsible for inhibiting pathogens’ growth. That being said, the three pure compounds had a different effectiveness in terms of function of the microorganisms, compared to the leaf extract (i.e., *Oomycota* growth did not halt completely when exposed to leaf extract, only slowed). We can hypothesize that depending on the molecules, the pathogen could inactivate and/or metabolize them. Similar outcomes regarding ITC effectiveness and usage is reported in the literature (Sung and Lee, 2007; Ugolini et al., 2014; de Melo Nazareth et al., 2019; Evangelista et al., 2021; Kwun et al., 2021).

Concerning the GSLs, we identified and quantified 15 compounds. GSV is the most abundant, and is normally found in rocket leaves (Bell and Wagstaff, 2019), and has been identified as the GSL with effectiveness towards fungi, bacteria etc. (Jaafar and Jaafar, 2019). Sweet Flame cv. was found to contain the highest amount of glucosativin, but no concentrations of sativin were observed. This suggests other non-GSL derived compounds present in leaves may be responsible for its significant activity towards *Pp. chamaehyphon*. GER was the second most abundant GSL compound quantified, as found in the literature (Stajnko et al., 2022), along with known anti-microbial activity (Kudjordjie et al., 2022). DGBT was the third most abundant, but there are no reports of its effectiveness against fungi in the literature. The remaining compounds were in a significantly lower quantity than the three mentioned. A positive correlation of the radial growth inhibition was observed for total GSLs, especially for *Pp. chamaehyphon* and *Pp. vexans*, while a weak and negative correlation was observed for *Phy. citrophthora*.

It is noteworthy that erucin was not detected in the cultivars Astra and Dentellata. Likewise, sativin was not detected in the Astra, Eruca 16, Sweet Flame, and Sweet Intensity cultivars. Victoria produced the highest concentrations for both erucin and sativin. Conversely, across the remaining cultivars where erucin and sativin were observed, no statistically significant deviations in values were observed among them. Furthermore, these values consistently remained lower when juxtaposed with the chemical concentrations exhibited by Victoria. The correlation between erucin and sativin in relation to the inhibition of radial growth in pathogens demonstrated an elevated level consistent effect in this instance. For *Pp. chamaehyphon* and *Pp. vexans*, the correlation coefficients exhibited a range between 0.58 and 0.78. An attenuated correlation was discerned in the case of *Phy. citrophthora*, denoting coefficients of -0.39 and 0.29. This noteworthy observation underscores that the ultimate downstream products, namely erucin and sativin, warrant primary consideration for their impact on the biocontrol of pathogens and their specificity. Their robust correlations with the inhibition of pathogenic radial growth support the hypothesis of their role in shaping the biocontrol efficacy (Zhu et al., 2020; Liu et al., 2021). Conversely, in the remaining cases, correlations were observed to be weak, displaying both positive and negative trends.

It is important to determine the potential effect of a host plant’s secondary allelochemicals, in this case, GSLs and downstream products, on the growth of plant pathogens *in vitro*, prior to it being applied under greenhouse or field conditions, to maximize the effectiveness. Our study is the first to explore the possibility of the biocontrol of KVDS, using rocket leaf extracts containing GSL, and GSL-derived products. Furthermore, our research indicates that this is an effective tool in reducing the growth of oomycetes pathogens (~67% average reduction in growth) yet being an environmentally friendly tool. It may be possible to use a combination of leaf extracts to improve their efficacy towards the *Oomycota*, thus strengthen their overall effectiveness. Confirmation of the effect of leaf extracts as fungal biopesticides will be assessed under field *in vivo* conditions in future research.

## 5 Conclusion

This study highlights the effectiveness of salad rocket and its GSL content and hydrolysis product formation in reducing the spread of candidate pathogens. Thus, the use of *Eruca* leaf extract, can be considered a promising biocontrol tool against KVDS. It was found to have an inhibitive effect on the growth of soil-borne pathogens. Additionally, the biochemicals found in the leaf extracts were identified and compared with a liquid chromatography–mass spectrometry (LC-MS) analysis to determine if there were any differences in their composition that could explain the pathogens’ growth inhibition. Data indicate that the presence of high levels of hydrolysis products (erucin and sativin) are an important component in counteracting growth of oomycetes, especially in some rocket lines. Although *in vitro* experiments are generally the first step in a long journey, this study forms the basis for further field investigations.

## Conflicts of interest

The authors declare that the research was conducted in the absence of any commercial or financial relationships that could be construed as a potential conflict of interest.

## Data availability statement

The original contributions presented in the study are included in the article/**Supplementary Material**. Further inquiries can be directed to the corresponding author.

## Author contributions

GM: Conceptualization, Data curation, Formal Analysis, Investigation, Methodology, Software, Validation, Visualization, Writing – original draft. KZ: Data curation, Investigation, Writing – review & editing. LB: Formal Analysis, Investigation, Writing – review & editing. SL: Formal Analysis, Investigation, Writing – review & editing. LB: Conceptualization, Data curation, Formal Analysis, Project administration, Supervision, Validation, Writing – review & editing. PE: Conceptualization, Funding acquisition, Project administration, Resources, Supervision, Writing – review & editing. GC: Conceptualization, Funding acquisition, Project administration, Resources, Supervision, Writing – review & editing.

## Supplementary material

Supplementary Figure 1 Example of study preparation of supernatant extracted **(A)** and Petri dishes with the pathogens’ plug **(B)**.

Supplementary Figure 2 Examples of Petri dishes from the study. On the right-hand side; *Phy. citrophthora*, whilst on left-hand side *Pp. vexans*.

Supplementary Figure 3 Examples of Petri dishes from the study. The pathogen into account is *Pp. vexans*.

Supplementary Figure 4 Examples of Petri dishes from the study. The pathogen into account is *Pp. chamaehyphon*.
