# Supplementary figures and images for: In vitro application of Eruca vesicaria subsp. sativa leaf extracts and associated metabolites reduces the growth of Oomycota species involved in Kiwifruit Vine Decline Syndrome
Source: Front Plant Sci. 2023 Dec 18;14:1292290. doi: 10.3389/fpls.2023.1292290 (PMC10757965; doi:10.3389/fpls.2023.1292290)

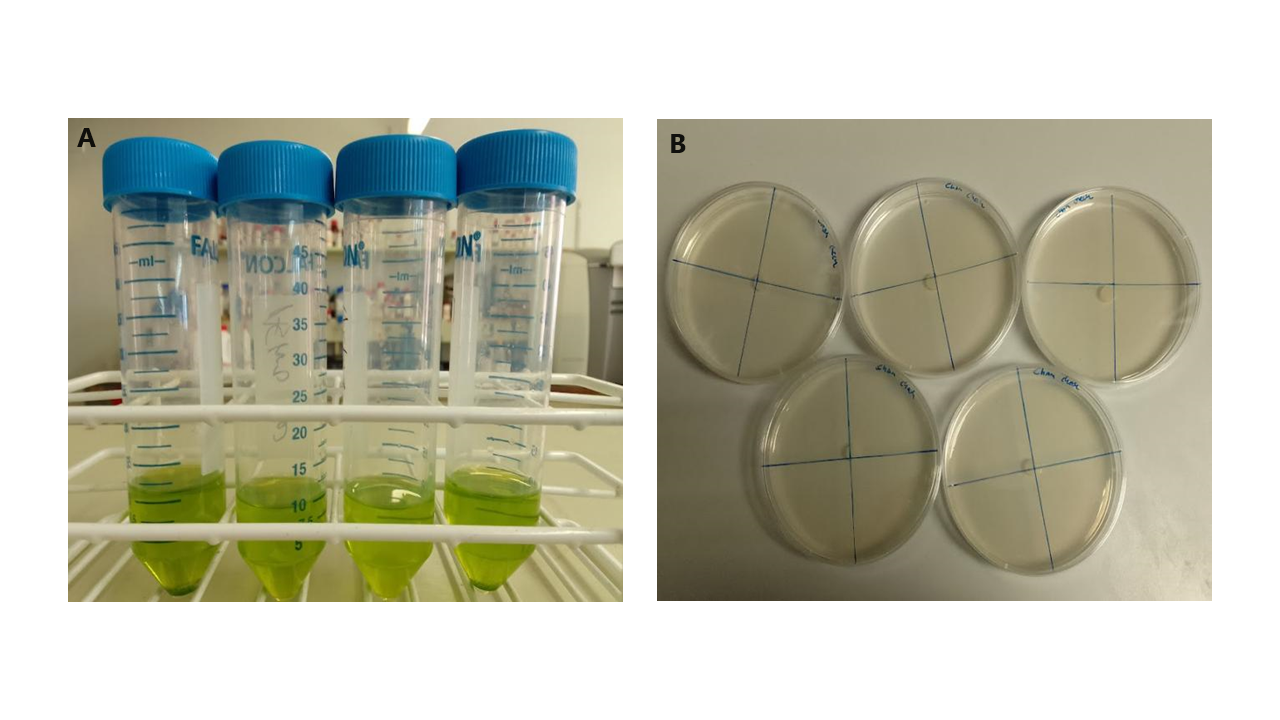

Supplement: Supplementary Figure 1 — Example of study preparation of supernatant extracted (A) and Petri dishes with the pathogens’ plug (B). [file Image_1.tif]

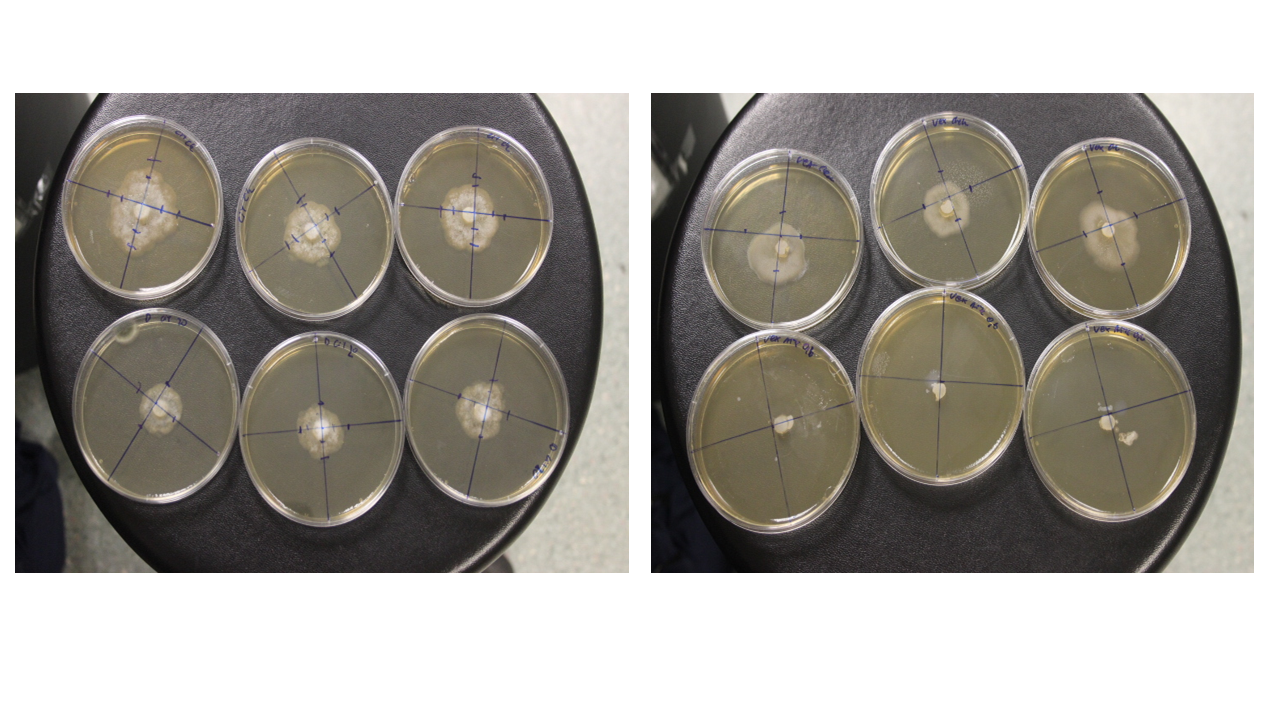

Supplement: Supplementary Figure 2 — Examples of Petri dishes from the study. On the right-hand side; Phy. citrophthora, whilst on left-hand side Pp. vexans. [file Image_2.tif]

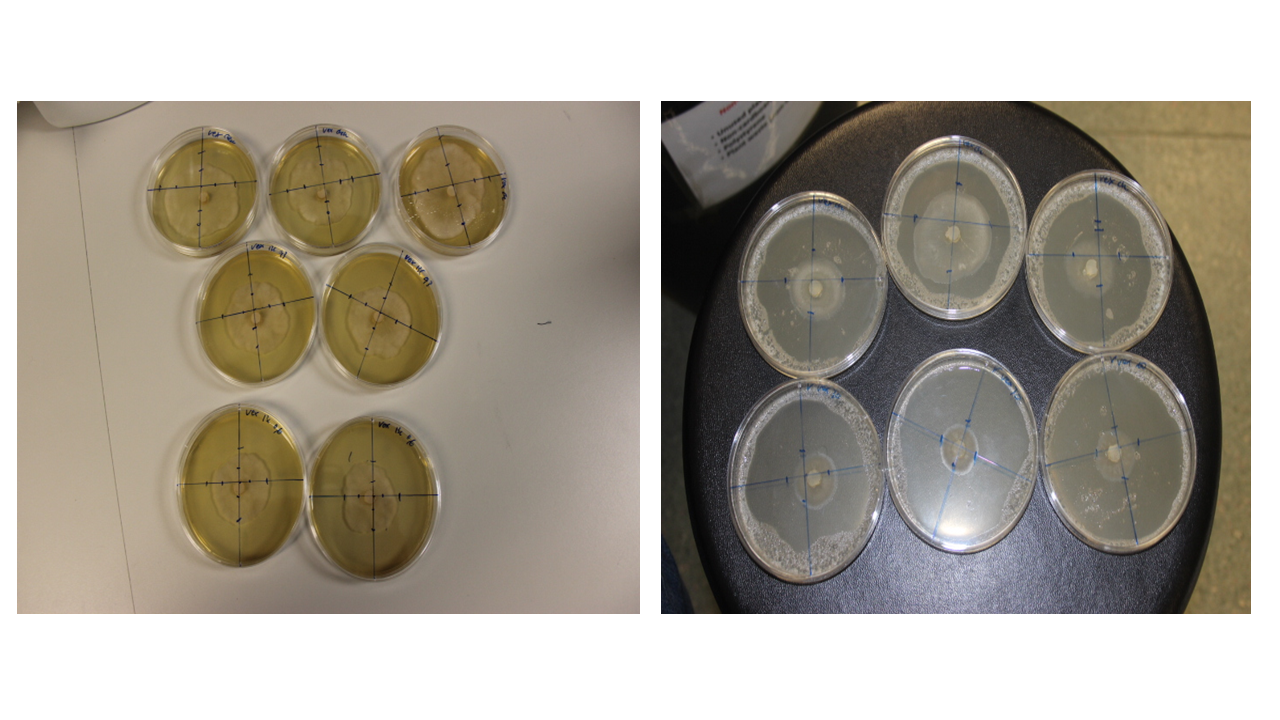

Supplement: Supplementary Figure 3 — Examples of Petri dishes from the study. The pathogen into account is Pp. vexans. [file Image_3.tif]

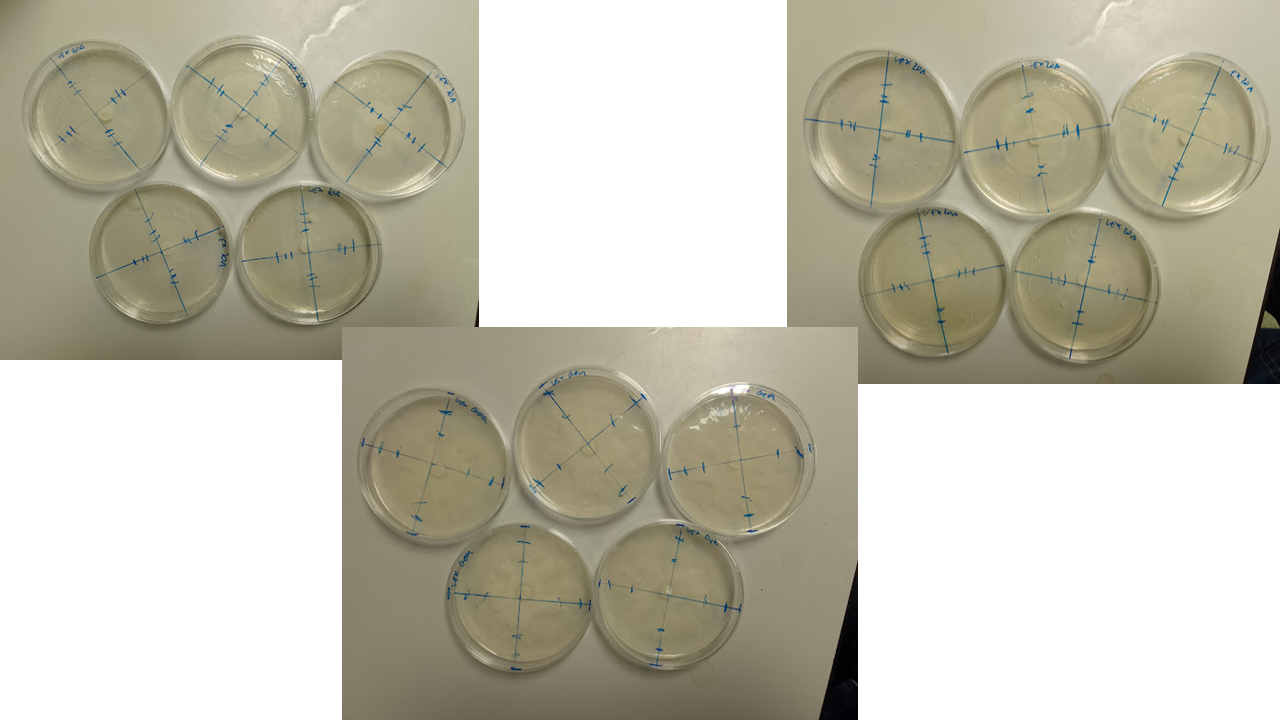

Supplement: Supplementary Figure 4 — Examples of Petri dishes from the study. The pathogen into account is Pp. chamaehyphon. [file Image_4.tif]
